# Scalp myiasis associated with soft tissue sarcoma lesion: a case report and review of relevant literature

**DOI:** 10.1186/s12879-023-08957-8

**Published:** 2024-01-05

**Authors:** Sahar Azarmi, Kamran Akbarzadeh, Ali Ekrami, Zahra Sheikh, Omid Dehghan

**Affiliations:** 1 Department of Medical Entomology and Vector Control, School of Public Health, Tehran University of Medical Sciences, Tehran, Iran; 2 Department of Nursing, University of Social Welfare and Rehabilitation Sciences, Tehran, Iran; 3 Department of Medical Parasitology and Mycology, School of Medicine, Iranshahr University of Medical Sciences, Iranshahr, Iran

**Keywords:** Fly, *Sarcophaga*, Sarcoma cancer, Myiasis, Iran

## Abstract

**Background:**

Sarcophagidae is one of the main fly families that is attracted to open wounds, ulcers, lesions, and other injuries for depositing their larvae. The presence of larvae of flies in human tissues makes myiasis. Myiasis on the scalp could be more frightening in comparison with myiasis on the other parts of the body. It is a rare myiasis case that shows the ability of myiasis agents to attack various parts of the body. On the other hand, reporting of myiasis cases by Sarcophagidae larvae is not common due to difficulties in their identification. This study aimed to emphasize the importance of Sarcohagidae larvae in producing myiasis by describing the first case of soft tissue sarcoma infestation and provides a review of human myiasis by larvae of the Sarcophagidae family during 2010–2023 and also a review of wound myiasis cases associated with malignancy during 2000–2023.

**Case presentation:**

A case of sarcoma cancer myiasis is reported on the scalp of a 43-year-old man who referred to one of Tehran’s hospitals for surgical treatment of cancer. Before surgery, insect larvae were observed in the area of ​​sarcoma. The larvae were isolated, examined morphologically, and identified as *Sarcophaga* spp.

**Conclusions:**

Myiasis has been considered as a neglected disease. Publishing of myiasis cases could be useful to alert health policy-makers about its danger and appearance in the community. It is not usual but can be expected even on the scalp of the human head. Exact daily supervision and dressing of the wound could be recommended to prevent cutaneous myiasis.

## Background

Myiasis is the parasitic infestation of humans with the larvae (maggots) of the dipteran flies which grow inside the host’s tissue and feed on it. Myiasis can affect different body parts, including the skin, eyes, ears, nose, mouth, and gastrointestinal tract [1]. Cutaneous myiasis is the most common clinical form depending on the site of involvement. Wound myiasis (traumatic myiasis) is the main clinical manifestation of cutaneous myiasis [2].

Myiasis can be caused by members of several fly families, such as blowflies (Calliphoridae), flesh flies (Sarcophagidae), botflies (Oestridae), and so on. Different species of mentioned fly families can cause different types of myiasis depending on the site and type of infestation [3].

In Iran, myiasis is one of the health problems, especially in rural areas with the high number of traditional animal husbandry places. A total of 26 human myiasis cases by different species of fly larvae were reported in Iran from 2013 to 2020 [4]. There was a published review of myiasis cases in Iran which reported 77 cases of various kinds of myiasis in Iran till 2014 [5]. The most common species were *Lucilia sericata* and *Chrysomya bezziana*, respectively [4, 5].

Different types of wound myiasis, which occur in different parts of the body, such as the head, face, and scalp, have been reported in Iran [6–9]. Skin cancer is one of the causes of chronic skin ulcers in humans. Persistent ulcers caused by skin cancer, including squamous cell carcinoma and basal cell carcinoma, provide a suitable substrate for myiasis [8, 10–12].

Sarcophagidae is one of the most important families of flies that is attracted to open wounds, ulcers, lesions, and other injuries for depositing their larvae. The larvae then feed on surrounding tissues, causing damage, and can produce a range of symptoms depending on the severity and site of the infestation [13]. The most common symptoms of cutaneous myiasis includes pain, itching, swelling, redness, skin breakdown and ulcers. Also, the open wound caused by the infestation with fly larvae can be infected with bacteria, leading to symptoms such as fever, discharge of pus, and increased pain and swelling [14].

For reviewing previous studies on human myiasis cases during 2010–2023, a search was conducted using the MeSH keywords such as “myiasis”, “Sarcophagidae”, and “sarcoma cancer” in the websites related to reputable medical journals such as PubMed, Google Scholar, Scopus, Web of Science, IranMedex, MagIran and ISC databases. More than 173 scientific sources published in English between 2010 and 2023 were collected. Then, irrelevant sources and articles were removed, and finally, 32 articles associated with myiasis by Sarcophagide flies were selected, interpreted and analyzed considering the purpose of the study (Table 1). Also, for reviewing recent articles on myiasis associated with cancerous wounds during 2000–2023, the MeSH keywords including “myiasis”, “larvae”, “scalp”, “cancer”, “carcinoma”, “ulcer”, and “sarcoma cancer” were checked in the mentioned scientific websites. In total, 45 articles were found, of which 25 cases related to the present study were considered for literature review (Table 2).

**Table 1:** Myiasis cases caused by larvae of the family Sarcophagidae during 2010–2023

Author	Year	Type of myiasis	Fly species	Country	Reference
Abdel-Hafeez et al.	2015	Cutaneous Myiasis	*Sarcophaga haemorrhoidalis*	Egypt	[2]
Ayalon A et al.	2020	Ophthalmic myiasis	*S. argyrostoma*	Israel	[15]
Ergün S et al.	2016	Cutaneous Myiasis	*S. carnaria*	Turkey	[1]
Martínez-Rojano H et al.	2018	Nasal Myiasis	*Sarcophaga *spp.	Mexico	[16]
De Pasquale R et al.	2019	Cutaneous Myiasis	*Sarcophaga* spp.	Italy	[17]
Ahmad AK et al.	2011	Gastrointestinal myiasis	*Sarcophaga* sp.	Egypt	[18]
Ly P et al.	2018	Intestinal Myiasis	*Sarcophaga* spp.	Peru	[19]
Giangaspero A et al.	2017	Cutaneous Myiasis	*S. argyrostoma*	Italy	[20]
Zhou M et al.	2021	Oral myiasis	*S. ruficornis*	China	[21]
Demirel Kaya F et al.	2014	Cutaneous Myiasis	*Sarcophaga* spp.	Turkey	[13]
Polat E et al.	2016	Middle ear myiasis	*Sarcophaga* sp.	Turkey	[22]
Najjari M et al.	2020	Gastrointestinal Myiasis	*S. argyrostoma*	Iran	[23]
Hiraoka H et al.	2015	Genital	*S. crassipalpis*	Japan	[24]
Severini F et al.	2015	Tracheostomy Myiasis	*S. argyrostoma*	Italy	[25]
Das A et al.	2010	Intestinal myiasis	*Sarcophaga* spp.	India	[26]
Ferraz AC et al.	2010	Cutaneous Myiasis	*S. ruficornis*	Brazil	[3]
Dutto M et al.	2011	Cutaneous Myiasis	*Sarcophaga* spp.	Italy	[27]
Zaglool D et al..	2013	Cutaneous Myiasis	*Sarcophaga* spp.	Saudi Arabia	[28]
Dutto M et al.	2010	Cutaneous myiasis	*S. cruentata*	Italy	[29]
Norouzi R et al.	2017	Intestinal myiasis	*Sarcophaga* spp.	Iran	[17]
Supreme HS et al.	2015	Intestinal myiasis	*Sarcophaga* spp.	Nepal	[30]
Jang H et al.	2022	Cutaneous Myiasis	*Sarcophaga* spp.	Korea	[31]
Subramanya SH et al.	2019	Intestinal myiasis	*S. peregrina*	China	[32]
Song S et al.	2016	Cutaneous Wound	*S. africa*	Korea	[22]
Wakid MH et al.	2022	Cutaneous Myiasis	*Wohlfahrtia magnifica*	Saudi Arabia	[33]
Martins LG et al.	2021	Cutaneous Myiasis	*S. ruficornis*	Brazil	[34]
Chiewchanvit S et al.	2017	Cutaneous Myiasis	*Sarcophaga* spp.	Thailand	[35]
Iqbal J et al.	2011	Cutaneous Myiasis	*Sarcophaga* spp.	Kuwait	[36]
Salimi M et al.	2010	Urogenital myiasis	*W. magnifica*	Iran	[37]
Withers, P. Roy, L	2010	Cutaneous Myiasis	*S. argyrostoma*	France	[38]
Tileklioğlu E et al.	2021	Cutaneous Myiasis	*Sarcophaga* spp.	Turkey	[39]

**Table 2:** Wound myiasis cases associated with malignancy during 2000–2023

Gender	Age	Year	Neoplasm	Localization	Fly species	Country	Reference
Female	85	2003	Basal cell carcinoma	Orbit	*Hypoderma bovis*		[40]
Female	90	2003	Squamous cell carcinoma	Eyelid and orbit	*Chrysomya bezziana*	Hong Kong	[41]
Male	80	2005	Squamous cell carcinoma	Eyelid	*Chochliomyia hominivorax*	India	[42]
Male	27	2006	Squamous cell carcinoma	Scalp	Unknown	USA	[43]
Male	54	2006	laryngeal carcinoma	Neck	*Chrysomya* spp.	Spain	[44]
Female	101	2006	Skin tumors	Scalp	*Sarcophaga* spp.	Spain	[44]
Female	87	2006	Skin tumors	Face	*Sarcophaga* spp.	Spain	[44]
Female	65	2007	Squamous cell carcinoma	Orbit	Unknown	USA	[45]
Male	41	2007	Epidermoid carcinoma	Penis	Unknown	Brazil	[46]
Male	69	2007	Basal cell carcinoma	Scalp	*Lucilia sericata*	Netherlands	[47]
Male	58	2007	Basal cell carcinoma	Periauricular area	Unknown	USA	[48]
Female	46	2008	Carcinoma of hypopharynx	Neck and pharynx	*C. bezziana*	India	[49]
Female	52	2008	Carcinoma of larynx	Tracheal stoma	*C. bezziana*	India	[49]
Male	62	2008	Carcinoma of cheek	Face	*C. bezziana*	India	[49]
Male	75	2008	Carcinoma of the lower lip	Lip	*C. bezziana*	India	[49]
Male	80	2008	Epidermoid carcinoma	Face	Unknown	Brazil	[50]
Female	72	2008	Squamous cell carcinoma	Face and neck	Unknown	Brazil	[51]
Male	64	2008	Tumor	Scalp and skull	Unknown	Turkey	[52]
Female	75	2009	Squamous cell carcinoma	Nose and eyeball	*L. sericata*	Japan	[53]
Male	61	2009	Squamous cell carcinoma	Oropharynx, head and neck	*Lucilia* spp.	Germany	[54]
Female	65	2009	Squamous cell carcinoma	Face	*C. bezziana*	Iran	[55]
Male	24	2011	Squamous cell carcinoma	Eyelid and orbit	family Calliphoridae	India	[56]
Male	70	2012	Squamous cell carcinoma	Auricle	Unknown	Iran	[57]
Female	64	2012	Basal cell carcinoma	Scalp	Unknown	Iran	[8]
Male	73	2012	Squamous cell carcinoma	Head and neck	*W. magnifica*	Turkey	[58]
Female	48	2014	Invasive ductal carcinoma	Breast	Unknown	India	[59]
Male	50	2015	Squamous cell carcinoma	Oral area and face	Unknown	India	[60]
Male	55	2015	Squamous cell carcinoma	Oral area and face	Unknown	India	[60]
Female	95	2016	Squamous cell carcinoma	Scalp	Unknown	USA	[11]
Male	60	2020	Squamous cell carcinoma	Extraoral region and the alveolar region	Unknown	Brazil	[61]
Male	elderly	2022	Squamous cell carcinoma	Scalp	*L. sericata*	Japan	[12]

Myisis has been considered as a neglected disease in all around the world [62]. Under reporting of myiasis cases, especially in the cases of nosocomial myiasis, is a common phenomenon [63]. Myiasis on the scalp could be more frightening in comparison with myiasis on the other parts of the body. It is a rare myiasis case that shows the ability of myiasis agents to attack various parts of the body. On the other hand, reporting of myiasis cases by Sarcophagidae larvae is rare due to difficulties in their identification. This study aimed to emphasize the importance of Sarcohagidae larvae in producing myiasis by describing the first case of soft tissue sarcoma infestation and provides a review of human myiasis by maggots of the Sarcophagidae family during 2010–2023 and also a review of wound myiasis cases associated with malignancy during 2000–2023.

## Case presentation

**Fig. 1:**
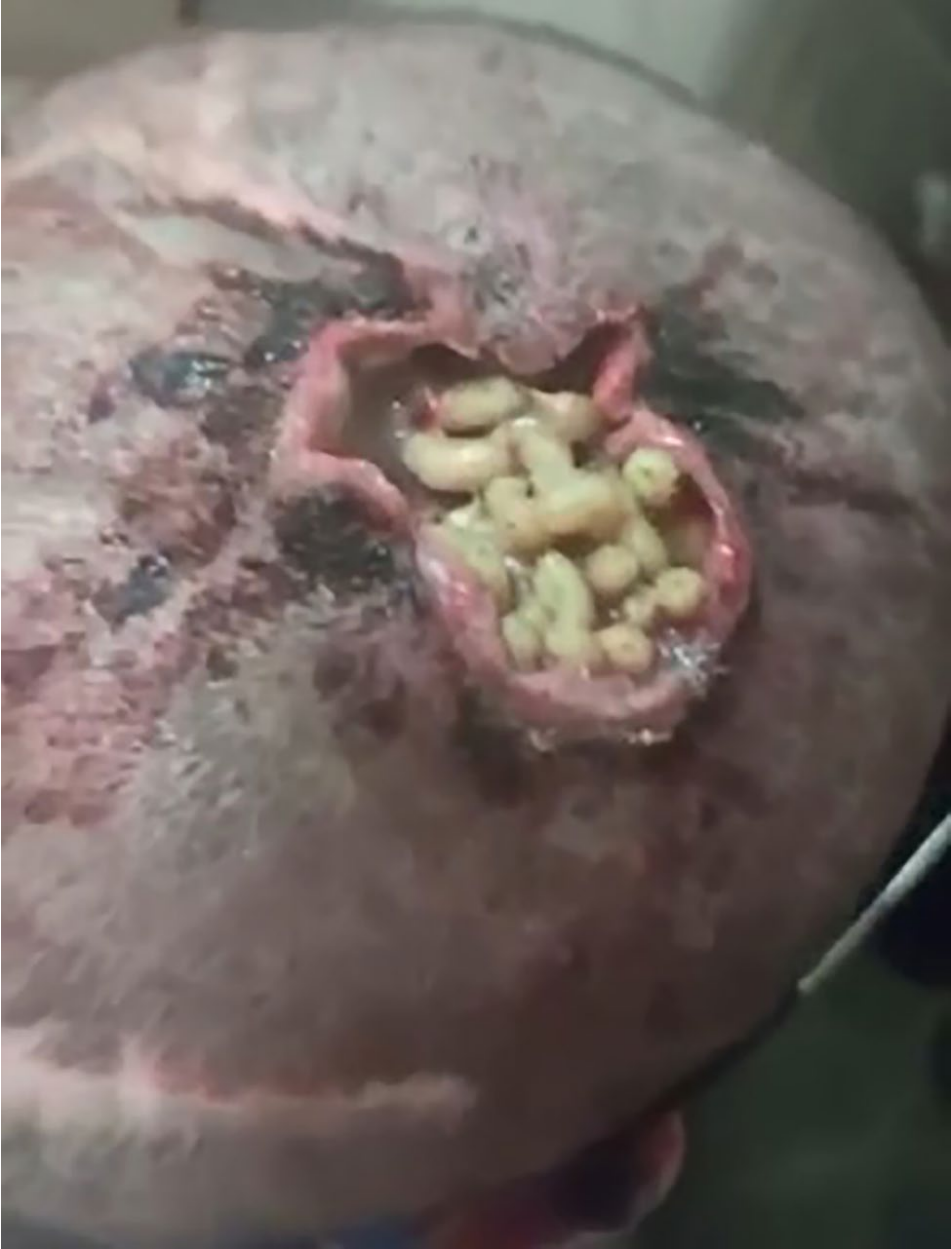
The larvae in a soft tissue sarcoma in the upper part of the head

A 43-year-old man from Qazvin province, Iran, referred to the Cancer Institute of Imam Khomeini Hospital in Tehran following complaints of progressive scalp ulceration (without bone involvement). Soft tissue sarcoma was the diagnosis made after completing the initial procedures and mailing the pathology sample, and he was admitted to the hospital for surgery in early 2023. The day before the surgery, when nurses shaved and trimmed the patient’s hair (which was about 10 cm) to prepare the scalp area, they noticed the presence of a large number of maggots on the wound (Fig. 1). The man was assessed and treated by the cancer surgery professors there. The patient did not have any risk factors, such as autoimmune disorders, that raise the chance of cancer, nor did he have a family history of cancer or genetic diseases. Additionally, he had reported having no history of Hypertension (HTN), hyperlipidemia (HLP), or diabetes mellitus (DM). In his biography, he mentioned smoking and alcohol consumption going back 10 years, and he omitted high-risk occupations like working in mines, textile and dyeing industries, or direct sunlight.

Upon further examination, the presence of the larvae was confirmed in the upper part of the head involved in soft tissue sarcoma. The maggots had eaten the lower part of the wound edge. Twenty-seven third-stage larvae were collected from the wound area with forceps, and the area was thoroughly washed with normal saline and bandaged. He underwent skin flapping from the leg area following the surgical excision of the malignant lesion. Following a partial recovery, he was discharged from the surgical department and directed to the radiotherapy and chemotherapy services to complete his treatment. The patient, who lived in a wealthy and luxurious area of Qazvin province, had no contact with animals. Also, he was completely unaware of the presence of larvae before observing these maggots in the wound and felt no movement of the maggots. It is necessary to mention that the patient is alive and continues his treatment.

**Fig. 2:**
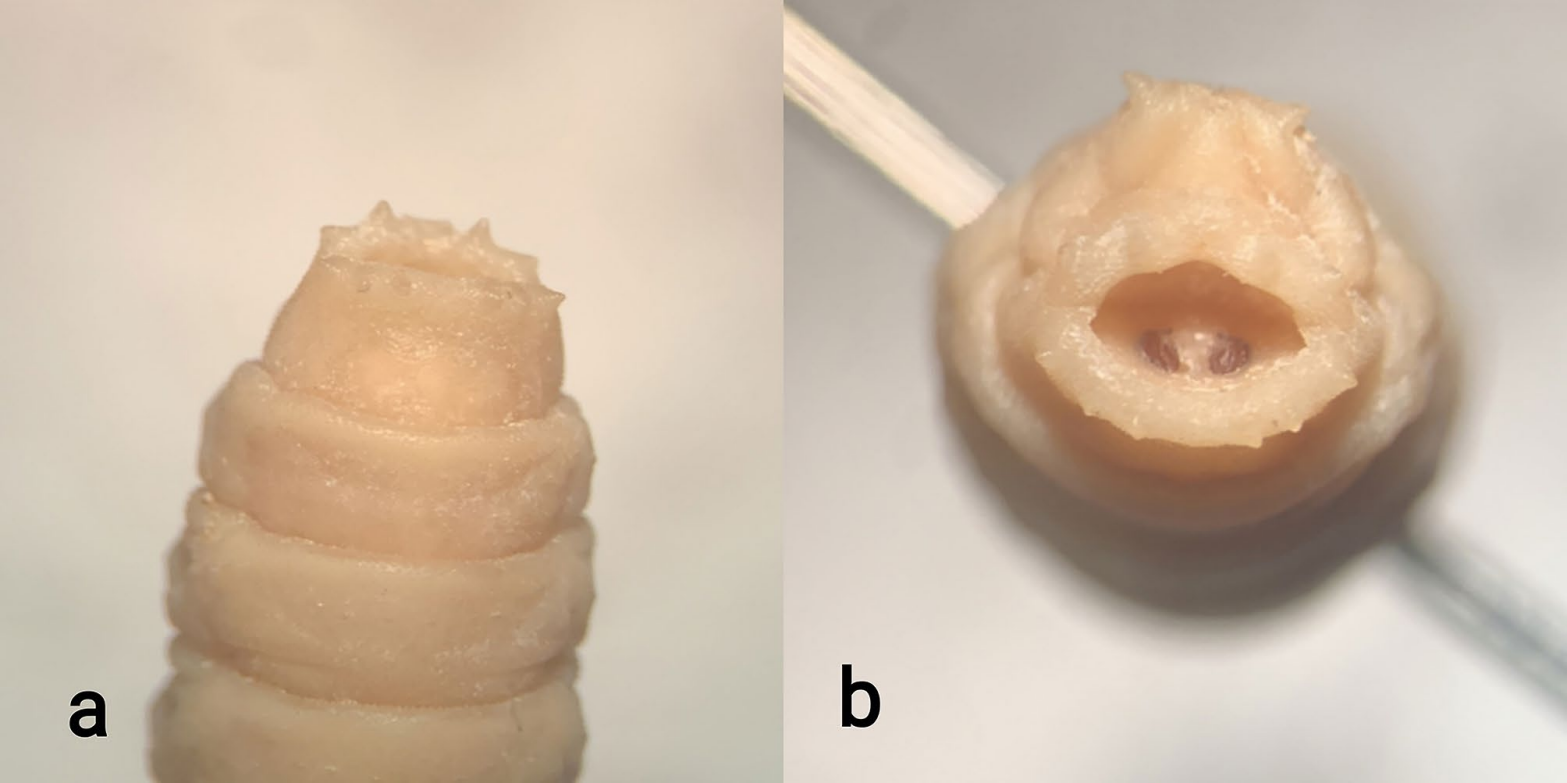
The *Sarcophaga* spp. larvae collected from the soft tissue sarcoma lesion of the scalp; **a**: spiracular cavity developed as a deep depression, and **b**: entrance of spiracular cavity broad

The larvae were transferred to the medical entomology lab at the Tehran University of Medical Sciences and identified as the *Sarcophaga* spp. (Diptera: Sarcophagidae). The situation of posterior spiracle that is hidden inside of the cavity, is the most important feature for identifying the *Sarcophaga* spp. larvae in which the slits run obliquely outwards or downwards (Fig. 2) [64]. This is the first report of soft tissue sarcoma infestation by maggots of the Sarcophagidae family.

## Discussion and conclusions

Myiasis is defined as the infestation of live vertebrates (humans and animals) tissues with dipterous larvae [65]. Although myiasis is infrequent in developed nations, it is prevalent in tropical and subtropical regions as well as areas with inadequate sanitation. The actual number of cases of this infection may be higher than what is reported due to it needing to be more adequately reported [66, 67].

Cutaneous myiasis is the most common form of myiasis in which flies lay eggs in necrotic, hemorrhagic and abscess-like lesions [2]. Based on the current study, most cases of wound myiasis over the past 13 years have been caused by flies of the families Sarcophagidae and Calliphoridae. Furthermore, cutaneous myiasis was the most frequently reported case of myiasis in the Sarcophagidae family (17 out of 31 cases) (Table 1).

Non-healing wound is one of the symptoms of skin cancer, such as squamous cell carcinoma, which is a suitable substrate for myiasis [12]. Soft tissue sarcoma is a rare malignancy that can develop from soft tissues such as fat, muscle, nerves, fibrous tissue, blood vessels or deep skin tissue. Soft tissue sarcoma can become infested if left untreated [68]. Wound myiasis of two major types of non-melanoma skin cancer, basal cell carcinoma and squamous cell carcinoma, caused by *L. sericata* and other species of fly larvae, have been documented in different parts of the world. Almost all patients were over 60 years old [8, 10–12, 44, 65, 69, 70] (Table 2). Rubio et al. (2006) reported three cases of myiasis in patients with tumor lesions. The first case was a 54-year-old man, and the other two cases, 101 and 87-year-old women, suffered from laryngeal carcinoma and skin tumors on the scalp and face (squamous cell carcinoma), respectively. The larvae collected from the first case were *Chrysomya* spp., while the larvae in ulcers of women were confirmed to be *Sarcophaga* spp. [44]. According to our literature review on myiasis associated with malignancy, most of the patients belonged to a low socioeconomic status from suburban areas.

Flies of the genus *Sarcophaga* are known to cause myiasis in necrotic wounds and in anatomical cavities where fluid has accumulated [3]. The current study also confirmed this issue. Based on previous studies, poor hygiene, poor social conditions, old age, diabetes, vascular occlusive disease [2, 15, 19, 27], mental retardation, alcoholism [71] and nevoid basal cell carcinoma syndrome [21, 22] are the main predisposing factors for myiasis. In addition, wounds with purulent secretions, blood and body secretions are the most common factors that attract female flies [3]. In the current report, soft tissue sarcoma and the presence of necrotic and infectious tissue were among the most important predisposing factors for infestation with larvae of fly.

Annually, cases of myiasis due to different species of flies are reported from different regions of Iran [4, 5, 9]. According to a review article, human myiasis has been reported in 16 out of the 31 provinces in Iran, with Fars Province accounting for over 62% of all reported cases [5]. Based on the review article by Jokar (2022), elderly people (> 60 years) are more susceptible to infection, and women and men have an equal chance of getting myiasis [4]. Also, this study [4] showed that most of the infested people lived in urban areas (85.5%), and a small percentage were related to rural areas (11.5%). The fly species *L. sericata* (26.9%) and *C. bezziana* (19.2%) were the most common. Alizadeh et al. (2014) confirmed that a total of 77 different types of myiasis cases had been identified in Iran before 2014, and the majority of cases (52%) were oral myiasis [5]. In contrast to the study of Jokar (2022) [4], Alizadeh et al. (2014) showed that the majority of patients fell within the age range of 21 to 40 years old, accounting for 41.2% of the total. However, there was also a noticeable number of individuals above the age of 61, and most of the myiasis cases were due to *Oestrus ovis* (Diptera: Oestridae) (65%) [5].

Ibrahim Kokcam and Cem Ecmel Saki reported a case of cutaneous myiasis caused by *Sarcophaga* spp. Larvae in a farmer with Nevoid Basal Cell Carcinoma Syndrome (NBCCS) with left frontotemporal pain, blood discharge and a necrotic ulcerative lesion with a hemorrhagic lesion [72]. De Pasquale (2019) reported myiasis caused by *Sarcophaga* spp. in a patient with cutaneous lymphoma on the surface of scalp lesions. At the clinical inspection, the patient showed lesions on various parts of the skinhead: plaques and scaly patches that appeared yellowish-green on an erythematous background, with a widespread location and cavities containing larvae underneath that which was finally were removed during curettage surgery [17].

Myisis is one of the neglected health issues in all around the world [62]. Under reporting of myiasis cases is a usual phenomenon, especially in the cases of nosocomial myiasis [63]. Despite of noticeable prevalence and appearance of various kinds of myiasis, the disease has no place to be recorded and reported in the health system of Iran. Reporting of myiasis cases could alert the health policy-makers about the presence of various kinds of myiasis in Iran. Suitable dressing could be recommended for any wounds in hospitals to prevent attacking of flies.

## Data Availability

All data and materials of this article are included in the manuscript.
